# Alteration of serotonin release response in the central nucleus of the amygdala to noxious and non-noxious mechanical stimulation in a neuropathic pain model rat

**DOI:** 10.1186/s12576-024-00910-x

**Published:** 2024-03-12

**Authors:** Ryota Tokunaga, Hideshi Shibata, Mieko Kurosawa

**Affiliations:** 1 Center for Medical Sciences, International University of Health and Welfare, Otawara, Tochigi 324-8501 Japan; 2 Laboratory of Veterinary Anatomy, Institute of Agriculture, Tokyo University of Agriculture and Technology, Fuchu, Tokyo 183-8509 Japan; 3 Bio-Laboratory, Foundation for Advancement of International Science, Tsukuba, Ibaraki 305-0821 Japan; 4 Present Address: Department of Physical Therapy, Niigata University of Health and Welfare, Niigata, 950-3198 Japan

**Keywords:** Neuropathic pain, Serotonin release, Central nucleus of the amygdala, Stroking, Pinching, Rat

## Abstract

Previously, we found that serotonin (5-HT) release in the central nucleus of the amygdala (CeA) of anesthetized rats decreases in response to innocuous stroking of the skin, irrespective of stimulus laterality, but increases in response to noxious pinching applied to a hindlimb contralateral to the 5-HT measurement site. The aim of the present study was to determine whether intra-CeA 5-HT release responses to cutaneous stimulation were altered in an animal model of neuropathic pain induced by ligation of the left L5 spinal nerve. In anesthetized neuropathic pain model rats, stroking of the left hindlimb increased 5-HT release in the CeA, whereas stroking of the right hindlimb decreased it. Meanwhile, pinching of the left hindlimb increased intra-CeA 5-HT release irrespective of stimulus laterality. In conclusion, the present study demonstrated that intra-CeA 5-HT release responses to cutaneous stimulation are altered in an animal model of neuropathic pain.

## Introduction

The amygdala is a major component of the limbic system and is well known to be critically involved in emotional processes, including very predominantly, in the induction of unpleasant emotions, such as fear. Neuroimaging studies have demonstrated that the amygdala is activated in human subjects experiencing an unpleasant feeling [1–3]. In concordance, amygdala activation in animals has been associated with the performance of anxiety- and fear-related behaviors [4–6].

Sensory stimulation of the skin or muscle can produce modality-dependent emotional changes. For example, noxious mechanical stimulation tends to evoke fear and anxiety, whereas innocuous tactile stimulation can be pleasant and anxiolytic. Serotonin (5-HT) in the central nucleus of the amygdala (CeA), has been shown to be involved in the triggering of anxiety and fear [7, 8]. Therefore, previously, we investigated how noxious and innocuous cutaneous stimulation affects 5-HT release in the CeA in anesthetized rats. In that study, we observed increases and decreases in 5-HT release in response to noxious (pinching) and innocuous (stroking) mechanical stimulation, respectively [9]. Those findings suggested that increases in 5-HT release in the CeA evoked by pinching may contribute to fear or anxiety, while decreases evoked by stroking may attenuate fear or anxiety in conscious animals.

The CeA has been implicated in the pathophysiology of chronic pain in human patients [10]. Neuroplastic changes in the CeA, including neural responses to somatic stimulation (both noxious and innocuous), were found to be augmented in an animal model of chronic arthritis pain [11]. Parabrachial tract stimulation-evoked excitatory synaptic transmission in the CeA has been shown to be enhanced in slice preparations from animal models of arthritis pain [12] and neuropathic pain [13].

The aforementioned results suggest that somatosensory stimulation-induced 5-HT release responses in the CeA may be altered by chronic pain. Thus, the aim of the present study was to analyze CeA 5-HT release in response to noxious and innocuous mechanical stimulation in an animal model of neuropathic pain. Release of 5-HT was measured with high-performance liquid chromatography (HPLC), and the noxious and innocuous forms of stimulation applied were pinching and stroking, respectively.

## Materials and methods

### Ethics

All experiments were conducted in accordance with the Japanese Physiological Society’s Guide for the Care and Use of Laboratory Animals. The study protocol was approved by the animal ethics committee of the International University of Health and Welfare.

### Animals

The experiments were performed on 13 male Wistar rats (280–330 g) obtained from Japan SLC, Inc (Shizuoka). The animals were kept in a temperature-controlled room (23 ± 1 °C) that was lit between 08:00 h and 20:00 h (Showa, Tokyo). Commercial rodent chow (Labo-MR stock, Nosan, Kanagawa) and tap water were provided ad libitum.

### Peripheral neuropathic pain model

The left L5 spinal nerve was ligated in isoflurane-anesthetized rats lying in a prone position as described in detail previously [14]. Briefly, from level L4 to level S2, we detached the paraspinal muscles from the spinous processes. Using, a small rongeur, we removed the L6 transverse process carefully, exposing the L4 and L5 spinal nerves. The left L5 nerve was then ligated firmly with 6-0 silk suture thread, and then the overlying surgical wound was sutured. Sham operations were performed on the right side as above with the omission of the nerve ligation step.

### Assessment of neuropathic pain development

Pain tests were performed with von Frey filaments (0.4–15 g, Stoelting, USA), as described by Chaplan et al. [15], before (day 0) and after (days 1, 3, 5, and 7) the L5 spinal nerve ligation operation. All tests were conducted between 18:00 h and 19:00 h. The criterion for the development of neuropathic pain was a 50% withdrawal threshold with Dixon’s up-down method [16].

### Cannula implantation

Each rat was implanted with a guide cannula (diameter: 0.5 mm; AG-12, Eicom, Kyoto) 1–2 d prior to the HPLC experiment as described in detail previously [9]. Microdialysis probe guide cannulae aimed at the CeA were positioned according to the following coordinates: 2.3 mm posterior to bregma, 4.0 mm lateral of the midline, and 6.4 mm ventral to dura.

### Microdialysis probe implantation and dialysate sampling

The experiments were conducted with rats anesthetized with urethane (1.1 g kg^−1^, intraperitoneal injection) 7 days after the spinal nerve ligation. The rats’ tracheas were intubated for spontaneous breathing, and their body temperatures were maintained at 37.5 ± 0.1 °C with heating pads and infrared lamps (ATB-1100, Nihon-Kohden, Tokyo). Depth of anesthesia was assessed routinely throughout the experiment by counting the number of breaths taken per minute and by testing corneal and flexion reflexes.

The microdialysis probe placement and dialysate sampling procedures were performed as described by Tokunaga et al. [9]. On the morning of the experiment day, a concentric microdialysis probe with a 1-mm membrane (220-μm outer diameter, 50-kDa molecular-weight cut-off; A-I-12–01, Eicom) was inserted into the CeA via the previously implanted guide cannula. The microdialysis probe was perfused at a rate of 1 µl min^−1^ with modified Ringer’s solution consisting of 147 mM Na^+^, 4 mM K^+^, and 1.15 mM Ca2^+^. Dialysate was collected from the microdialysis outlet tube for a period of 10 min. In vitro 5-HT recovery rates for individual probes ranged from 7.7 to 12.4%. To standardize recovery rate, 5-HT concentrations in the dialysate were calculated based on the assumption of a 10.0% recovery rate.

### HPLC

For 5-HT release measurements, pooled dialysate samples were injected manually into an HPLC chromatograph equipped with an electrochemical detector (HTEC-500, Eicom), as described previously [9]. For a standard 0.06 fmol μl ^−1^ solution, a 0.95% coefficient of variation was obtained with this method (*n* = 8).

### Cutaneous stimulation

Noxious mechanical stimulation, in the form of pinching, and innocuous mechanical stimulation, in the form of stroking, were applied as described in our previous study [9]. Briefly, pinching was applied with a surgical clamp (an area of 1.5 cm^2^) to the unilateral hindlimb (between the iliac crest and knee joint) at a force of 3–5 kg for 10 min. To avoid accommodation to the pinching, the stimulus site was changed every 2 min within the hindlimb, reaching a total pinching area of approximately 7.5 cm^2^. Stroking was applied manually to the hindlimb (an area of approximately 7–8 cm^2^) with a pressure of 80–100 mN cm^−2^ for 10 min at a frequency of 65–75 strokes min^−1^ (1.08–1.25 Hz). To avoid the influence of the skin tissue damage caused by pinching, stroking was always applied before pinching.

### Probe placement verification

At the conclusion of the experiment, the rats were anesthetized deeply with sodium pentobarbital. After transcardial perfusion with formalin, performed as described previously [9], each rat’s brain was removed and stored in the same fixative. Sections (50 µm thick) were cut with a freezing microtome, mounted, stained with thionin, and viewed under a light microscope.

### Statistical analysis

Data are expressed as means ± standard deviations (SDs). To detect changes over time within a group, we conducted repeated measures one-way analyses of variance (ANOVAs) followed by Dunnett’s multiple range post hoc tests. Experimental sets (responses in the left vs. right CeA; responses to stimulation of the ipsilateral vs. contralateral hindlimb) were compared with two-way ANOVAs. Prestimulus basal intra-CeA 5-HT concentration values were compared between experimental sets with Student’s *t*-tests. Probability values of less than 5% were considered significant.

## Results

### Probe placement

The probes were confirmed to be in the CeA for all rats used in this study.

### Withdrawal threshold

**Fig. 1:**
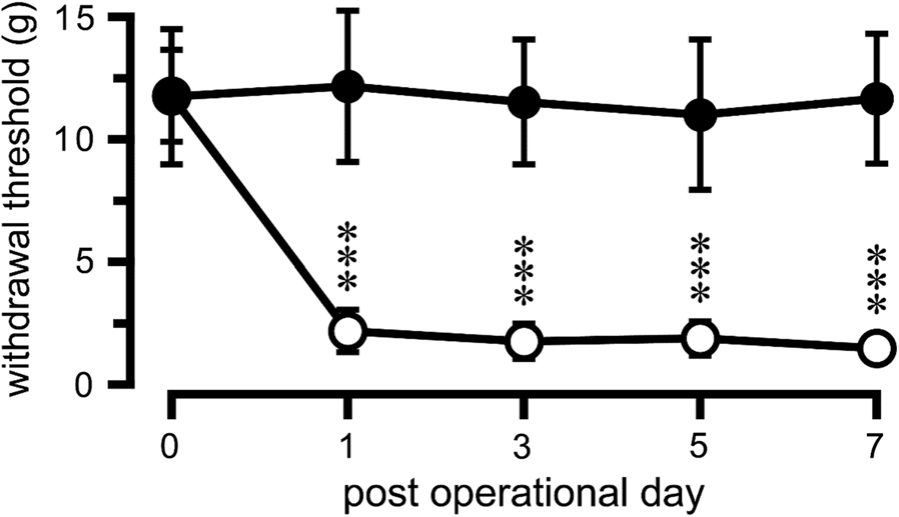
Changes in pain threshold after left L5 nerve ligation. Ordinate: 50% paw withdrawal threshold. Abscissa: days after ligation. Day 0 was the day of ligation. Closed circles, withdrawal of the right paw (non-ligated side); open circles, withdrawal of the left paw (ligated side); ***p < 0.001 vs. pre-ligation value (day 0). *n* = 13

The withdrawal thresholds evoked by stimulation of the right and left hindpaw with von Frey filaments were 12.5 ± 2.5 g and 12.1 ± 1.6 g (*n* = 13), respectively, in rats before ligation of the left L5 nerve (day 0 values shown in Fig. 1). The threshold of the left hindpaw (ligation side) decreased dramatically to 2.3 ± 0.9 g on day 1 after the ligation, whereas that of the right hindpaw (non-ligation side) remained unchanged over the week after the ligation operation (Fig. 1). The withdrawal thresholds observed for the right and left hindpaws on the experimental day (7 days after ligation) were 11.8 ± 2.6 g and 1.5 ± 0.4 g, respectively. The withdrawal thresholds were similar between experiments involving the right CeA and those involving the left CeA (data not shown).

### Responses to stroking stimulation

#### 5-HT release in the right CeA

**Fig. 2:**
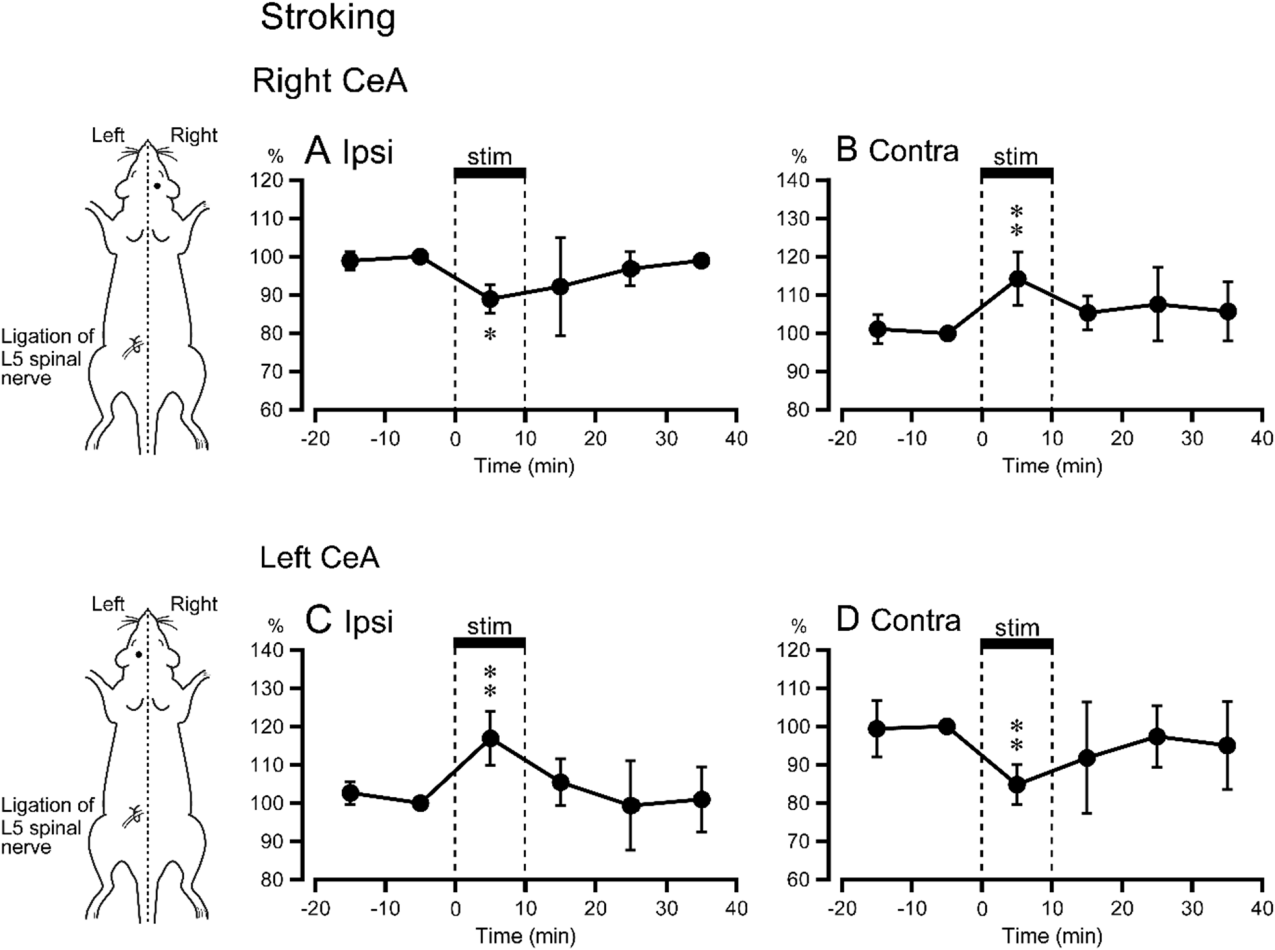
Release of 5-HT in the CeA in response to stroking stimulation of the hindlimb. Ordinates: response magnitude expressed as a mean percentage (± SD) of the prestimulus control value. Abscissas: time, with stimulation onset at 0 and the 10-min stimulus period shown with a horizontal bar. **A**, **B** The right CeA, *n* = 6. **C**, **D** The left CeA, *n* = 6. **p < 0.01, *p < 0.05 vs. prestimulus control values

Prestimulus control 5-HT release values in the right CeA obtained prior to stroking of the right (1.19 ± 0.37 fmol⋅10^–1^ min, *n* = 6) and left (0.99 ± 0.36 fmol⋅10^–1^ min, *n* = 6) hindlimb were similar. All data of right and left stimulation were obtained from the same 6 rats. When stroking stimulation was applied to the right hindlimb (non-ligated side) ipsilateral to 5-HT measurement, 5-HT release was significantly decreased during the stimulation period (89 ± 4% of prestimulus control values) (Fig. 2A). Stroking stimulation of the left hindlimb (ligated side), contralateral side to the measurement, produced significant increases in 5-HT release (114 ± 7% of prestimulus control values) during the stimulation period (Fig. 2B).

#### 5-HT release in the left CeA

Prestimulus control 5-HT release values in the left CeA did not differ significantly between right hindlimb stroking (1.09 ± 0.62 fmol 10^–1^ min, *n* = 6) and left hindlimb stroking (1.21 ± 0.52 fmol 10^–1^ min, *n* = 6) conditions. Of the six data, five were obtained from the same animals for both left and right stimulation. The remaining data were obtained from separate animals.

Stroking of the left hindlimb (ligated side, ipsilateral to the measurement) and of the right hindlimb (non-ligated, contralateral to the measurement) produced significant increases (117 ± 7 of prestimulus control values) and decreases (85 ± 5 of prestimulus control values) in left-CeA 5-HT release, respectively (Fig. 2C, D). The magnitudes of these increased and decreased intra-left CeA 5-HT release responses to stroking of the left (Fig. 2C) and right (Fig. 2D) hindlimb, respectively, were similar to those observed for the analogous experiments reported above for the right CeA (Fig. 2A, B).

### Responses to pinching stimulation

#### 5-HT release in the right CeA

**Fig. 3:**
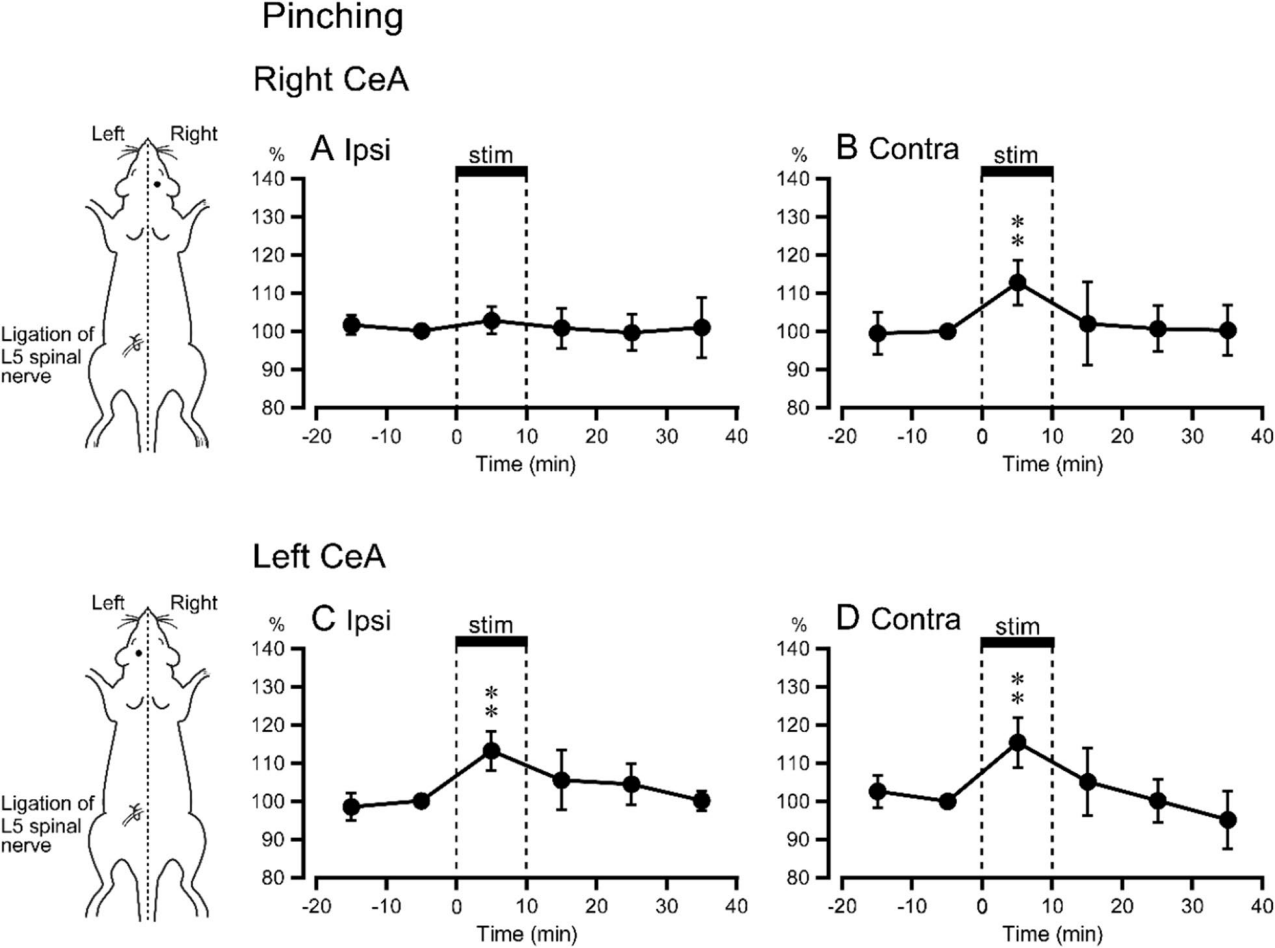
Release of 5-HT in the CeA in response to pinching stimulation of the hindlimb. **A**, **B** The right CeA, *n* = 5. **C**, **D** The left CeA, *n* = 5. **p < 0.01 vs. prestimulus control values. Axes are as in Fig. 2

Prestimulus control 5-HT release values in the right CeA obtained prior to right hindlimb pinching (1.11 ± 0.45 fmol 10^–1^ min, *n* = 5) and prior to left hindlimb pinching (1.02 ± 0.42 fmol 10^–1^ min, *n* = 5) were similar to each other. Of the five data, four were obtained from the same animals for both left and right stimulation. The remaining data were obtained from separate animals. When pinching stimulation was applied to the right hindlimb (non-ligated side, ipsilateral to the measurement), 5-HT release remained similar to prestimulus levels over the 40-min observation period (Fig. 3A). On the other hand, pinching stimulation of the left hindlimb (ligated side, contralateral to the measurement) produced significant increases in intra-right CeA 5-HT release during the stimulation period (113 ± 6% of prestimulus control values) (Fig. 3B).

#### 5-HT release in the left CeA

Prestimulus control 5-HT release values in the left CeA obtained prior to right hindlimb pinching (1.04 ± 0.77 fmol 10^–1^ min, *n* = 5) were statistically similar to those obtained prior to left hindlimb pinching (1.06 ± 0.57 fmol 10^–1^ min, *n* = 5). All data of right and left stimulation were obtained from the same 5 rats. When pinching was applied to the left hindlimb (ligated side, ipsilateral to the measurement), 5-HT release during the stimulation period increased to 113 ± 5% of prestimulus control values (Fig. 3C). Pinching stimulation applied to the right hindlimb (non-ligated, contralateral to the measurement) also increased 5-HT release (115 ± 7% of prestimulus control values) during stimulus period (Fig. 3D). The magnitudes of these increases in response to pinching of the left (Fig. 3C) and right (Fig. 3D) hindlimbs were similar.

The magnitudes of increased intra-left CeA 5-HT release in response to pinching of the left and right hindlimbs (Fig. 3C, D) were not different from intra-right CeA 5-HT release responses to pinching of the left hindlimb (Fig. 3B). Furthermore, the magnitudes of increased responses to pinching (Fig. 3B-D) were not significantly different from those to stroking of the left hindlimb (Fig. 2B and C).

## Discussion

The present study showed for the first time that 5-HT release responses to noxious and innocuous cutaneous stimulation in the CeA determined by HPLC in rat models of neuropathic pain were different from those seen previously in normal control animals [9]. These results suggest that neuroplastic changes altering 5-HT release in the CeA occur in our animal model of neuropathic pain. In a previous study involving normal (nerve-intact) rats, we found that innocuous stroking of the skin reduced 5-HT release in both the contralateral and ipsilateral CeA [9].

In the present study with rats given a left L5 nerve ligation, this 5-HT reduction effect was replicated bilaterally only for stimulation of the right hindlimb situated on the side without nerve ligation. On the contrary, stimulation of the left hindlimb, on the side with a ligated L5 nerve, resulted in increased 5-HT release in the CeA of our animal models of neuropathic pain. These new results demonstrate that innocuous hindlimb stimulation ipsilateral to L5 nerve ligation increases 5-HT release, whereas the same stimulation of on the non-ligated side decreases 5-HT release in both the right and the left CeA. Given that noxious pinching stimulation increases 5-HT release in intact animals [9], these results indicate that stroking stimulation of the hindlimb ipsilateral to the ligated nerve may mimic the effects of a noxious stimulation on the CeA, perhaps via a peripheral mechanism similar to that underlying allodynia [17–19]. Since L5 nerve injury is known to cause sensitization of fast-conducting high-threshold (group III) afferents [20, 21], stroking stimulation may evoke activation of group III afferents in  spinal nerve-ligated model rats. In addition, optogenetic stimulation of group II fibers has shown to induce paw withdrawal and activate neurons in the CeA in a model of peripheral nerve injury [22]. Therefore, activation of both group II and group III fibers may contribute to the response of 5HT release in the CeA to stroking stimulation of the ligated hindlimb.

Corticotropin releasing factor (CRF) neurons in the CeA project to the dorsal raphe nucleus (DRN) and, conversely, 5-HT neurons in the DRN project to the CeA [23]. In a previous study, we found that the differing 5-HT release responses to noxious and innocuous stimulation could be attributed to the contributions of different subtypes of CRF receptors in the DRN. That is, increased and decreased 5-HT release responses were found to be mediated via CRF2 and CRF1 receptors, respectively, in the DRN [24]. Furthermore, it has been reported that activity of 5-HT neurons in the DRN decreased following intra-DRN injection of a lower amount of CRF via CRF1 receptors but increased following intra-DRN injection of a higher amount of CRF via CRF2 receptors [25]. It is possible that stroking on the non-ligated side triggers a relatively low amount of CRF release into the DRN, resulting in stimulation of CRF1 receptors, which would decrease 5-HT release in the CeA. On the other hand, stroking on the ligated side may trigger a higher amount of CRF release in the DRN, resulting in stimulation of CRF2 receptors, which would increase 5-HT release in the CeA.

Nociceptive information reaches the CeA through the spino-parabrachio-amygdaloid pathway, which originates from lamina I neurons in the contralateral spinal cord [26–28]. Because innocuous stroking of the side ipsilateral to nerve ligation increased 5-HT release in the CeA measured on either side, it is unlikely that the spino-parabrachio-amygdaloid pathway participates in this response alteration. Alternatively, the CeA receives polymodal information from thalamic and cortical areas through the basolateral amygdaloid (BLA) [6, 29, 30]. Hence, given that there are bilateral corticothalamic projections [31] and corticothalamic pathway are driven even under anesthetic condition [32], the thalamus–BLA–CeA pathway may contribute to postligation CeA responses to stroking of either side.

However, regarding noxious stimulation, our prior observation that contralateral, but not ipsilateral, pinching is effective for increasing 5-HT release in the CeA of intact animals suggests that the spino-parabrachio-CeA pathway may contribute to increases in 5-HT release in response to pinching [9]. Consistent with the results obtained in intact animals, here, we found that 5-HT release in the right CeA of animals with a left L5 nerve ligation also increased only when the contralateral (left, ligated side) hindlimb was stimulated. On the other hand, in the left CeA experiments, increases in 5-HT release were observed in response to pinching of either the ipsilateral (left, ligated side) or contralateral (right, non-ligated) hindlimb. Taken together, pinching delivered to the ligated side increased 5-HT release in the CeA irrespective of laterality. Thus, the increased 5-HT release responses to ipsilateral pinching observed in rats with spinal nerve ligation may be mediated via the same pathway that conveys stroking information to the CeA, namely, the thalamus–BLA–CeA pathway.

Most of the 5-HT efferents to the CeA originate from the DRN [33–35], noxious stimuli induced a transient elevation of Ca^2+^ signals of DRN-CeA 5-HT neurons [36], and the firing rates of 5-HT neurons in the DRN have been reported to be elevated 7 d after spinal nerve ligation in rats [37]. Thus, increases in DRN neuronal firing could underlie augmented 5-HT release in the CeA of animals with spinal nerve ligation. In accordance, basal release of 5-HT in the CeA in the present nerve ligation animal model (0.98–1.21 fmol·10 μl^−1^) tends to be augmented compared to that observed in intact animals (0.84– 0.92 fmol·10 μl^−1^) in our previous study [9], though direct comparison is difficult across separate studies. Given the established involvement of 5-HT within the CeA in fear and anxiety-related behaviors [7, 8], augmented intra-CeA 5-HT release responses following spinal nerve ligation might lead to elevation of anxiety in neuropathic pain model animals.

Chronic pain-induced plasticity in the CeA has been reported to be dominant in the right hemisphere. For example, responses of neuronal activity to somatosensory stimulation were elevated in the right, but not the left, CeA after inflammation affecting either knee in an animal model of arthritis pain [38]. Additionally, the numbers of cells expressing c-Fos and phosphorylated extracellular signal-regulated kinases were increased after unilateral injection of formalin into either hindpaw in an inflammatory pain model in only the right CeA [39]. In the present study, we did not observe a laterality difference in release of 5-HT in response to hindlimb stimulation, indicating that 5-HT neurons projecting from the DRN to the CeA do not have right hemisphere dominance.

## Conclusions

The main finding of this study is that intra-CeA release responses to cutaneous stimulation are altered in an animal model of neuropathic pain, relative to those in intact animals [9]. Notably, stroking stimulation delivered to the side on which the L5 nerve was ligated resulted in increased 5-HT release responses in the CeA, as opposed to the decreased responses that occur in intact animals. Furthermore, pinching stimulation delivered to the ligated side showed increased 5-HT release responses irrespective of laterality, in contrast to the clear laterality observed in intact animals. The present data suggest that increased 5-HT responses to cutaneous stimulation, particularly in the CeA, may lead to augmented anxiety under chronic pain conditions.

## Data Availability

The datasets used and/or analyzed during the current study are available from the corresponding author on reasonable request.
